# Improved Heterojunction Quality in Cu_2_O-based Solar Cells Through the Optimization of Atmospheric Pressure Spatial Atomic Layer Deposited Zn_1-x_Mg_x_O

**DOI:** 10.3791/53501

**Published:** 2016-07-31

**Authors:** Yulia Ievskaya, Robert L. Z. Hoye, Aditya Sadhanala, Kevin P. Musselman, Judith L. MacManus-Driscoll

**Affiliations:** ^1^Department of Materials Science and Metallurgy, University of Cambridge; ^2^Cavendish Laboratory, University of Cambridge

**Keywords:** Chemistry, Issue 113, Cuprous oxide, Atmospheric pressure spatial ALD, ZnO/Cu_2_O heterojunction, inorganic solar cell, ZnO, interface recombination

## Abstract

Atmospheric pressure spatial atomic layer deposition (AP-SALD) was used to deposit n-type ZnO and Zn_1-x_Mg_x_O thin films onto p-type thermally oxidized Cu_2_O substrates outside vacuum at low temperature. The performance of photovoltaic devices featuring atmospherically fabricated ZnO/Cu_2_O heterojunction was dependent on the conditions of AP-SALD film deposition, namely, the substrate temperature and deposition time, as well as on the Cu_2_O substrate exposure to oxidizing agents prior to and during the ZnO deposition. Superficial Cu_2_O to CuO oxidation was identified as a limiting factor to heterojunction quality due to recombination at the ZnO/Cu_2_O interface. Optimization of AP-SALD conditions as well as keeping Cu_2_O away from air and moisture in order to minimize Cu_2_O surface oxidation led to improved device performance. A three-fold increase in the open-circuit voltage (up to 0.65 V) and a two-fold increase in the short-circuit current density produced solar cells with a record 2.2% power conversion efficiency (PCE). This PCE is the highest reported for a Zn_1-x_Mg_x_O/Cu_2_O heterojunction formed outside vacuum, which highlights atmospheric pressure spatial ALD as a promising technique for inexpensive and scalable fabrication of Cu_2_O-based photovoltaics.

**Figure:**
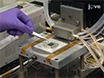


## Introduction

Cuprous oxide (Cu_2_O) is an earth-abundant non-toxic p-type semiconductor material^1^. With a band gap of 2 eV, cuprous oxide can fulfill the role of light absorber in heterojunction or tandem solar cells. In heterojunction solar cells, Cu_2_O is known to be paired with a variety n-type large band gap semiconductors such as ZnO^2^ and its doped variations^3,4^, Ga_2_O_3_^5,6^ and TiO_2_^7^ (For a more detailed overview on Cu_2_O photovoltaics see Ref.^8^). The development of Cu_2_O based heterojunction solar cells is presented in **Figure 1**, where the method of synthesizing the heterojunction is indicated next to each data point. One can note that vacuum based methods such as pulsed laser deposition (PLD) or atomic layer deposition (ALD) allowed for higher power conversion efficiencies to be achieved (up to 6.1%^9^). In contrast, the efficiencies for non-vacuum synthesis methods such as electrochemical deposition (ECD) have remained low. However, for low-cost photovoltaics it is better to synthesize the heterojunction outside a vacuum. While a vacuum-free, scalable technique of heterojunction formation is a more suitable alternative, it remains challenging to produce an interface of high quality by such methods. In this work we utilize an open-air, scalable thin film deposition process called atmospheric pressure spatial atomic layer deposition (AP-SALD) to grow n-type oxides for Cu_2_O-based solar cells. The advancement of AP-SALD over conventional ALD is that in the former, precursors are separated in space rather than in time^10^. During the deposition process, a substrate oscillates back and forth on a heated platen under a gas manifold which contains precursor gas channels separated by inert gas channels, as shown in **Figure 2**. The nitrogen gas carrying the precursors flows vertically through the gas manifold down towards the laterally moving platen. Due to the oscillations of the platen, each point on the substrate is sequentially exposed to the oxidant and metal precursors, as illustrated in **Figure 2**. This allows the metal oxide film to grow layer by layer. A detailed description of AP-SALD reactor design and operation can be found elsewhere.^11,12^ This approach allows the deposition to occur one to two orders of magnitude faster than conventional ALD and outside vacuum, which is compatible with roll-to-roll processing. High quality conformal oxide films produced by AP-SALD can be deposited at low temperatures (<150 °C) on a variety of substrates including plastics, which enables AP-SALD films to be applied to low-cost functional devices such as solar cells^13^, light emitting diodes^14^ and thin-film transistors^15^.

The custom made AP-SALD gas manifold used in this work was mechanically maintained over the substrate placed on the platen. This allowed control of the substrate-manifold spacing independent of the gas flow rates. A large spacing of 50 µm was used, which resulted in intermixing between the metal precursor and oxidant in the gas phase. Therefore, the AP-SALD reactor was operated in chemical vapor deposition (CVD) mode. This was found to be advantageous over operating in ALD mode because the films were grown at a higher rate, but still with high thickness uniformity and were crystalline when deposited at the same temperatures as ALD films.^12^ Herein, we still refer to the reactor as an AP-SALD reactor because it has the same fundamental design principles as other AP-SALD reactors.^11^

We used our reactor to deposit the n-type layer for our solar cells, in particular zinc oxide and zinc magnesium oxide (Zn_1-x_Mg_x_O^16,17^). Incorporating Mg into ZnO allows the conduction band to be tuned, which is important for reducing losses due to band-tail thermalization^13^ and interfacial recombination.^18,19^

Here we show how tuning the conditions for depositing zinc oxide and zinc magnesium oxide films on thermally oxidized cuprous oxide substrates allowed for improved interface quality and hence better solar cell performance to be obtained. This improvement was made possible through the identification of the major limiting factor in Cu_2_O based solar cells: recombination at the heterojunction interface due to an excessive formation of cupric oxide (CuO) on the Cu_2_O surface.

## Protocol

### 1. Preparation of Cuprous Oxide Substrates

1. **Oxidation of copper foil**
  1. Cut 0.127 mm thick Cu foil into 13 mm x 13 mm squares and clean by sonicating in acetone.
  2. Heat up copper foil to 1,000 °C while continuously flowing Ar gas through the furnace. Monitor the gas ambient in the furnace with a gas analyzer throughout the oxidation. When the temperature of 1,000 °C is reached, introduce oxygen to the furnace at a flow rate to obtain 10,000 ppm oxygen partial pressure and keep that for at least 2 hr. After 2 hr, turn off the oxygen but keep the Ar gas flowing.
  3. Cool down the furnace to 500 °C (keep the Ar gas flowing). Quench the oxidized samples by rapid withdrawal of the crucibles from the furnace. Dip the substrates into deionized water to cool them faster.
2. **Etching of Cu_2_O**
  1. Etch one side of the substrates by repeatedly applying a drop of dilute nitric acid (1:1 mixture of H_2_O and 70% HNO_3_) to remove any cupric oxide from the surface. Continue etching until no grey film is visible on the Cu_2_O surface. CAUTION: This procedure is performed in a fume hood.
  2. Immediately after etching, rinse each substrate in deionized water and sonicate in isopropanol. Dry with an air gun.
  3. Deposit 80 nm of gold onto the etched side of the Cu_2_O substrates by evaporating a 1 g gold pellet placed in a tungsten boat inside a resistance evaporator. Use base pressure 8 x 10^-6^ mbar and current of 4 A to reach the evaporation rate of 0.8 Å/sec.
  4. Etch the other side of the substrates in dilute nitric acid by applying a drop of acid onto the surface. Make sure the acid does not etch the golden film on the other side. Rinse and sonicate as described in section 1.2.2.
  5. Cover the substrates with a black insulating paint (use high temperature engine enamel) by using a paint brush, leaving an unmasked area of approximately 0.1 cm^2^ as the active area of the solar cell. Cover the golden electrode on the back side with a marker pen completely.

### 2. Depositing Zn_1-x_Mg_x_O Using AP-SALD Reactor

Note: Deposit Zn_1-x_Mg_x_O films on the unmasked side of Cu_2_O substrates.^13^ In this work, a custom-made AP-SALD reactor was used, adapted from the original design developed by Kodak.^11,12^ Details of the reactor customization are given in Ref. 12.

1. Set-up the AP-SALD system as follows:
  1. Use diethylzinc (DEZ) as the Zn precursor and bis(ethylcyclopentadienyl)magnesium as the Mg precursor. These are liquid precursors each contained in their separate glass bubblers. The precursors are pyrophoric and should never come in contact with air or water. The deposition system is gas-tight.
  2. For zinc oxide deposition, adjust the bubbling rate of nitrogen gas through the diethylzinc to 25 ml/min, which is contained at RT (20 °C). For zinc magnesium oxide deposition, adjust the gas fraction of each precursor by setting the bubbling rate through the diethylzinc to 6 ml/min and 200 ml/min through the bis(ethylcyclopentadienyl)magnesium (which is heated to 55 °C) to control to Zn to Mg ratio in the Zn_1-x_Mg_x_O.
  3. Set the flow rate of the nitrogen carrier gas for the metal precursor mixture to 100 ml/min. Bubble nitrogen gas at 100 ml/min through deionized water, which is employed as the oxidant. This vapor is diluted with nitrogen carrier gas flowing at 200 ml/min.
  4. Flow nitrogen gas at 500 ml/min to the gas manifold. In the AP-SALD gas manifold, this nitrogen gas is split to four separate channels. Each channel serves to spatially separate the two oxidant channels from the metal precursor mix channel between them.
2. Keep the gas manifold at a temperature of 40 °C via circulating water. Heat up the stage (moving platen) to the desired temperature (50 - 150 °C).
3. Set the desired sample-to-head distance, sample size, platen speed (50 mm/sec) and number of oscillations (cycles) with the software controlling the platen. The ZnO deposition rate is 1.1 nm/sec (or per cycle) and the Zn_1-x_Mg_x_O deposition rate is approximately 0.54 nm/sec at 150 °C. A typical number of deposition cycles is 200.
4. Deposit the desired oxide on a glass slide for 400 oscillations or until a clear thick homogeneous film can be seen.
5. Place the substrate on a glass mask if required, then place it under the gas manifold. Adjust the head (gas manifold) height to 50 µm above the substrate.
6. Deposit the Zn_1-x_Mg_x_O films by first opening the valves for the Mg precursor bubbler, then the Zn precursor bubbler, then start moving the platen under the gas manifold by clicking "start deposition" in the software. Open the H_2_O bubbler only after scanning the substrate with 5 oscillations of metal precursors in order to avoid Cu_2_O surface exposure to the oxidant while heated.
7. When the deposition is finished, remove the Cu_2_O substrates from the heated platen as quickly as possible and close the bubbler valves of the metal precursors. Clean the gas channels in the manifold with a blade to remove any deposited oxide powder. Start the next deposition cycle as described in 2.6.
8. When finished, purge the system for 30 min before closing the nitrogen valves.

### 3. Sputtering of ITO

1. Sputter 175 nm of indium tin oxide (ITO) by direct current magnetron sputtering^20^ at the following conditions: power 20 W, base pressure <10^-9^ mbar, Ar pressure 2.5 Pa. At a sputtering rate of 35 nm/min, sputter ITO for 5 min for a 175 nm thick ITO film. The resulting ITO/ZnO/Cu_2_O heterojunction is shown in **Figure 3**.

### 4. Finishing of the Devices

1. Clean the marker pen from the gold electrode with acetone to expose the golden electrode.
2. Apply electrical contacts by sticking 2 thin wires with Ag paste onto the ITO and Au electrodes.

## Representative Results

Thermodynamically, CuO is the only stable phase of copper oxide in air at RT, as the Cu-O phase stability diagram reveals^21^^-^^23^. To verify the presence of CuO on the surface of Cu_2_O, absorption spectra of the etched and unetched thermally oxidized Cu_2_O substrates were taken with photothermal deflection spectroscopy (PDS) — a highly sensitive technique which allows for sub-band gap absorption measurement^24^ (**Figure 4**). Both spectra showed absorption above 1.4 eV, which coincides with the band gap of CuO, before saturating at 2 eV (Cu_2_O band gap). The unetched substrate had a higher absorption below 2 eV, suggesting a thicker layer of CuO on the surface of unetched Cu_2_O than on the etched substrate. The inset in **Figure 4** shows a grey layer of CuO on the as-oxidized (unetched) Cu_2_O substrate. While no grey film could be detected visually on the etched substrate, some CuO was still present on its surface, as the PDS measurements suggests. The presence of a very thin CuO film on the surface of Cu_2_O substrates was also confirmed with x-ray photoelectron spectroscopy (XPS)^19,25^. Cupric oxide present on the Cu_2_O surface introduces deep level trap states (Cu^2+^)^18^ at the heterojunction interface that can act as recombination centers and, therefore, CuO presence at the p-n junction is undesirable.

Heating Cu_2_O substrates in the presence of oxidants (*e.g.*, air and moisture) facilitates the oxidation of Cu_2_O to CuO. In order to obtain polycrystalline ZnO by AP-SALD, the substrates are heated to 150 °C. As the substrate is held at elevated temperature in open-air or under the oxidant gas during the deposition, CuO quickly forms on the Cu_2_O surface. **Figure 5** shows scanning electron microscopy (SEM) images of an etched Cu_2_O substrate before and after spending 3 min on the AP-SALD platen at 150 °C under the flow of nitrogen. Multiple CuO outgrowths can be seen on the annealed substrate, with their composition being close to that of CuO as verified by energy-dispersive X-ray spectroscopy (EDX).

Photovoltaic devices were made with ZnO deposited by AP-SALD at 150 °C for 400 sec on top of the etched thermally oxidized Cu_2_O substrates. **Figure 6A** shows the surface of this standard device. One can notice numerous rod- and flower-like outgrowths present in the device. As confirmed earlier with EDX and PDS, these outgrowths are cupric oxide and occur due to Cu_2_O exposure to air and oxidants. **Table 1** and **Figure 7** ('ZnO/Cu_2_O standard' curve) demonstrate the relatively poor performance of this device.

In order to avoid CuO formation on the Cu_2_O surface, the conditions for depositing ZnO by AP-SALD on the etched thermally oxidized Cu_2_O substrates were optimized. The following measures were taken in order to minimize CuO growth: reduction of deposition temperature (**Figure 8A**); reduction of deposition time (**Figure 8B**); scanning the substrate surface for a few oscillations without exposure to the oxidant gas, *i.e.*, with only metal precursors and inert channels open (**Figure 8C**); and finally, avoidance of unnecessary heating of naked Cu_2_O substrates in air just before the start of deposition. The optimal parameters of ZnO deposition on Cu_2_O were found to be 100 °C, 100 sec and 5 water-free cycles. The surface of the optimized device was free of CuO outgrowths, as is demonstrated in **Figure 6B**. The current density–voltage (J-V) characteristic of the optimized ZnO/Cu_2_O device is compared with the standard device in **Figure 7**. The photovoltaic performance of both standard and optimized ZnO/Cu_2_O devices is presented in **Table 1**. It can be seen that by following the four above-mentioned measures, a six-fold increase in power conversion efficiency of the devices was achieved.

To further elucidate the effect of optimization of AP-SALD conditions on the reduction of CuO and the heterojunction quality, external quantum efficiency (EQE) measurements were performed on devices with ZnO deposited at 150 °C and 100 °C (**Figure 9**). The EQE spectra of the two devices, while similar at wavelengths above 475 nm, differed significantly at wavelengths below 475 nm, which is the range of wavelengths absorbed close to the interface. For the shorter wavelength radiation, the EQE of the device with ZnO made at higher temperature was less than half that of the device with ZnO made at lower temperature. This suggests that more cupric oxide was present at the ZnO/Cu_2_O interface made at higher temperature, which reduced charge collection from the region close to the heterointerface due to increased recombination.

Mg was incorporated into AP-SALD ZnO films in order to raise the conduction band of ZnO and to reduce recombination further^15^. Zn_1-x_Mg_x_O/Cu_2_O solar cells were made with the optimized Zn_0.8_Mg_0.2_O films, resulting in 2.2% device PCE — the highest to date for Cu_2_O-based solar cells with open-air fabricated heterojunctions (see the device performance in **Figure 7** and **Table 1**).


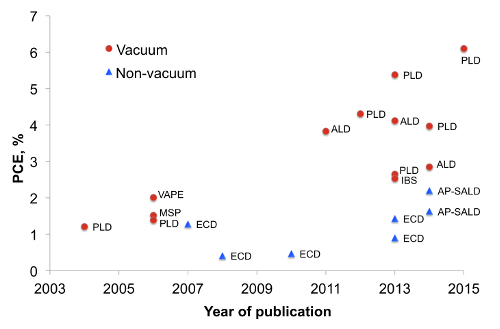
**Figure 1.Cu_2_O-based solar cell efficiency by year of publication (This figure has been modified from Ref.**^8^**). **Markers indicate whether the interface was formed in vacuum or in atmosphere (non-vacuum) and labels indicate the method of heterojunction formation. MSP - magnetron sputtering, IBS - ion beam sputtering, VAPE - vacuum arc plasma evaporation. Please click here to view a larger version of this figure.


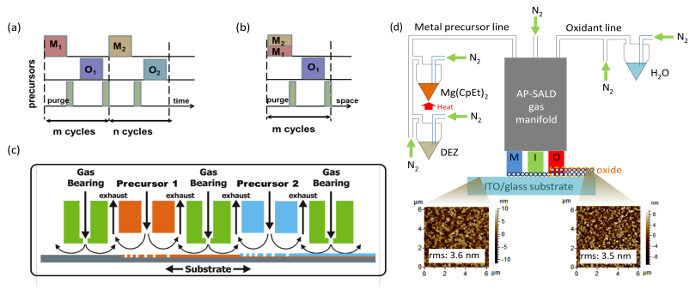
**Figure 2. Schematic of AP-SALD deposition process (compared with conventional ALD) and set-up for producing multicomponent metal oxides.** (**A**) Sequential exposure of each precursor and purge step in conventional ALD (delta-doping) (This figure has been reproduced from Ref. ^11^). In the context of this manuscript, M1 is diethylzinc vapor, M2 bis(ethylcyclopentadienyl)magnesium vapor, and O1 and O2 water vapor. (**B**) Sequential exposure of metal precursor mixture (co-injection), inert gas channels (equivalent to 'purge' step) and oxidant in AP-SALD (This figure has been reproduced from Ref. ^11^). (**C**) Schematic of a general AP-SALD reactor, showing the precursors spatially separated by inert gas channels, with the substrate oscillated beneath the different channels (This figure has been reproduced from Ref.^11^, which is a modification from one in Ref.^26^). (**D**) Overview schematic of the important components of an AP-SALD system with atomic force microscopy (AFM) images showing the morphology of the substrate before and after Zn_1-x_Mg_x_O deposition (This figure has been reproduced from Ref. ^13^). Please click here to view a larger version of this figure.


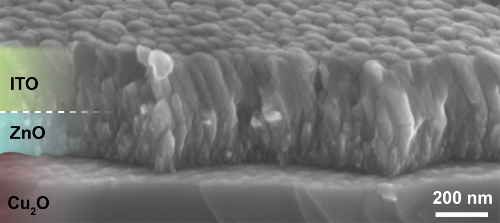
**Figure 3. Cross-sectional SEM image of ITO/ZnO/Cu_2_O heterojunction (This figure has been reproduced from Ref.^8^).** Conformal coating of Cu_2_O substrate with ZnO and ITO films can be observed. Please click here to view a larger version of this figure.


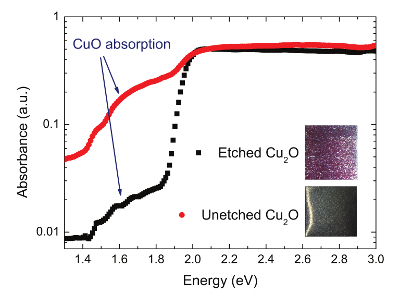
**Figure 4. PDS spectra of etched and unetched (as-oxidized) Cu_2_O substrates (This figure has been modified from Ref.^8^). **The insets show photographs of the etched and unetched cuprous oxide substrates. Please click here to view a larger version of this figure.


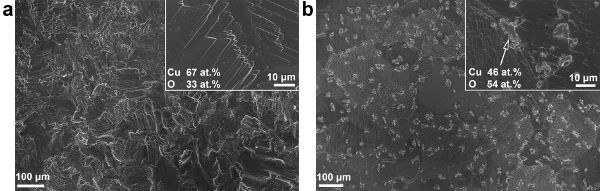
**Figure 5. SEM images of the surface of a Cu_2_O substrate when (A) freshly etched and (B) after annealing at 150 °C in air for 3 min (This figure has been reproduced from Ref.^8^). **Insets show surface composition acquired with EDX. Please click here to view a larger version of this figure.


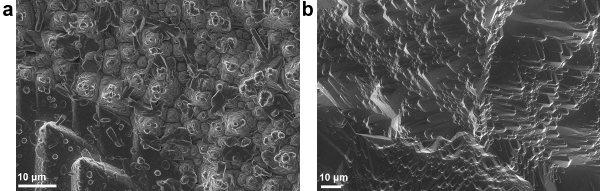
**Figure 6. SEM images of the surface of ZnO/Cu_2_O solar cells made using (A) standard conditions and (B) optimized conditions of AP-SALD ZnO (This figure has been reproduced from Ref.^8^). **Various outgrowths can be seen in the standard device. Please click here to view a larger version of this figure.


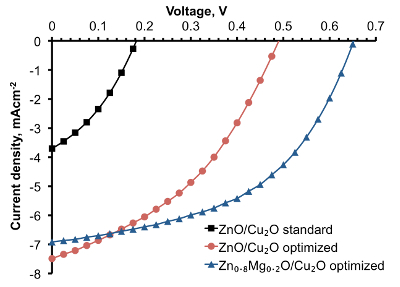
**Figure 7. Light J-V characteristics for Zn_1-x_Mg_x_O/Cu_2_O solar cells fabricated at standard and optimized AP-SALD conditions (This figure has been modified from Ref.^8^). **The J-V curves demonstrate solar cell performance improvement when the composition and AP-SALD conditions of the Zn_1-x_Mg_x_O films are optimized. Please click here to view a larger version of this figure.


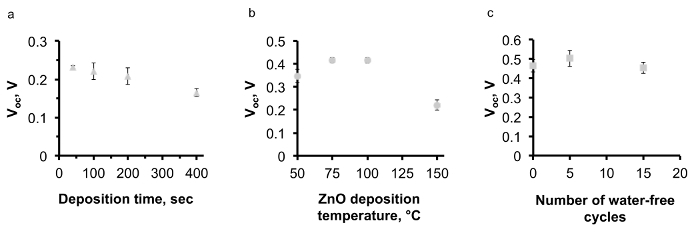
**Figure 8. The effect of AP-SALD parameters on the performance of ZnO/Cu_2_O solar cells. (A)** and **(B)** The effect of AP-SALD ZnO deposition time and temperature on the open-circuit voltage (V_oc_) of the devices (This figure has been reproduced from Ref.^8^), **(C)** correlation of water-free cycles with the V_oc_ of the devices. Please click here to view a larger version of this figure.


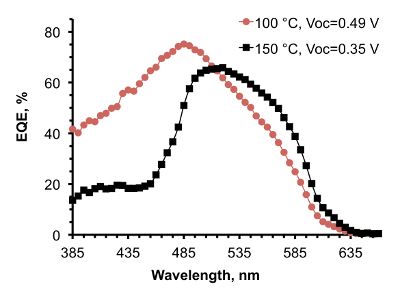
**Figure 9. EQE spectra of ZnO/Cu_2_O solar cells with ZnO deposited at 100 °C and 150 °C. (This figure has been reproduced from Ref.^8^). **Open-circuit voltage of the devices is indicated in the legend. Please click here to view a larger version of this figure.

**Table:** 

**Solar cell**	**Deposition temperature, °C**	**Deposition time, sec**	**J_sc_, mA/cm^2^**	**V_oc_, V**	**FF, %**	**PCE, %**
ZnO/Cu_2_O Standard	150	400	3.7	0.18	35	0.23
ZnO/Cu_2_O Optimized	100	100	7.5	0.49	40	1.46
Zn_0.8_Mg_0.2_/Cu_2_O Optimized	150	100	6.9	0.65	49	2.20

**Table 1. Standard and optimized AP-SALD Zn_1-x_Mg_x_O deposition parameters and performance of the best corresponding ITO/Zn_1-x_Mg_x_O/Cu_2_O solar cells (This table has been modified from Ref.**^8^**). **J_SC_- short circuit current density, FF - fill factor.

## Discussion

Critical steps within the protocol are stipulated by the Cu_2_O to CuO substrate surface oxidation. These include etching of the substrates in dilute nitric acid to remove any CuO after oxidation as well as after the evaporation of the golden electrode, minimizing the time substrates spend in open air before the Zn_1-x_Mg_x_O deposition and finally, deposition of Zn_1-x_Mg_x_O on Cu_2_O substrates by AP-SALD.

The advantage of AP-SALD compared to conventional ALD is that films can be grown outside a vacuum with a growth rate that is one to two orders of magnitude higher. However, this implies that the Cu_2_O substrates have to be exposed to oxidants in air at elevated temperature at least just before the deposition, which is sufficient for a thin CuO layer to form on the surface. This seemingly limits the application of the AP-SALD method to some oxidation-sensitive materials. However, by optimizing AP-SALD conditions such as temperature and time, as well as minimizing Cu_2_O exposure to air and moisture, a six-fold increase in the conversion efficiency of ZnO/Cu_2_O devices made using AP-SALD was achieved. The improvement came from the understanding that Cu_2_O to CuO oxidation is the major limiting factor of copper oxide as a material in heterojunction solar cells and modifying the fabrication protocol accordingly.

In order to completely avoid oxidation of cuprous oxide, the substrates have to be kept in an inert atmosphere or in vacuum all the time, which can be challenging when employing an open-air deposition technique such as AP-SALD. While the oxidation of Cu_2_O is avoided in vacuum based techniques^3,18^, for large-scale manufacturing, it is important that this problem can be minimized in atmospheric fabrication processes. In AP-SALD, the substrate surface can be exposed to reducing agents prior to the formation of the heterointerface, and by balancing the oxidation of Cu_2_O with the reduction of CuO by using forming gas during the deposition of the n-type oxide.^25^ The reducing agent used in AP-SALD could be a mixture of an inert gas with a reducing gas (*e.g.,* N_2_ + 5% H_2_^25^), or a number of cycles with a reducing precursor prior to the deposition, *i.e., *water-free cycles, in order to reduce CuO back to Cu_2_O just before the ZnO oxide starts to grow on its surface.

In this work, a standard protocol has been developed that minimizes CuO formation optimizing fabrication steps from Cu_2_O processing and etching to p-n junction formation by AP-SALD in open-air. The success of this work demonstrates the potential of AP-SALD as a promising method for application in cheap and scalable photovoltaic devices. The technique can be used for fast deposition of a variety of n- and p-type semiconducting metal oxides as well as blocking, buffer and barrier layers in solar cells on heat-sensitive substrates including plastics.

## Disclosures

The authors declare that they have no competing financial interests.
